# An ensemble-based machine learning solution for imbalanced multiclass dataset during lithology log generation

**DOI:** 10.1038/s41598-023-49080-7

**Published:** 2023-12-07

**Authors:** Mohammad Saleh Jamshidi Gohari, Mohammad Emami Niri, Saeid Sadeghnejad, Javad Ghiasi‑Freez

**Affiliations:** 1 Department of Petroleum Engineering, Kish International Campus, University of Tehran, Tehran, Iran; 2 Institute of Petroleum Engineering, School of Chemical Engineering, College of Engineering, University of Tehran, Tehran, Iran; 3 Department of Petroleum Engineering, Faculty of Chemical Engineering, Tarbiat Modares University, Tehran, Iran; 4 Faculty of Mining, Petroleum, and Geophysics, Shahrood University of Technology, Shahrood, Iran

**Keywords:** Geophysics, Crude oil

## Abstract

The lithology log, an integral component of the master log, graphically portrays the encountered lithological sequence during drilling operations. In addition to offering real-time cross-sectional insights, lithology logs greatly aid in correlating and evaluating multiple sections efficiently. This paper introduces a novel workflow reliant on an enhanced weighted average ensemble approach for producing high-resolution lithology logs. The research contends with a challenging multiclass imbalanced lithofacies distribution emerging from substantial heterogeneities within subsurface geological structures. Typically, methods to handle imbalanced data, e.g., cost-sensitive learning (CSL), are tailored for issues encountered in binary classification. Error correcting output code (ECOC) originates from decomposition strategies, effectively breaking down multiclass problems into numerous binary subproblems. The database comprises conventional well logs and lithology logs obtained from five proximate wells within a Middle Eastern oilfield. Utilizing well-known machine learning (ML) algorithms, such as support vector machine (SVM), random forest (RF), decision tree (DT), logistic regression (LR), and extreme gradient boosting (XGBoost), as baseline classifiers, this study aims to enhance the accurate prediction of underground lithofacies. Upon recognizing a blind well, the data from the remaining four wells are utilized to train the ML algorithms. After integrating ECOC and CSL techniques with the baseline classifiers, they undergo evaluation. In the initial assessment, both RF and SVM demonstrated superior performance, prompting the development of an enhanced weighted average ensemble based on them. The comprehensive numerical and visual analysis corroborates the outstanding performance of the developed ensemble. The average Kappa statistic of 84.50%, signifying almost-perfect agreement, and mean F-measures of 91.04% emphasize the robustness of the designed ensemble-based workflow during the evaluation of blind well data.

## Introduction

Recognizing lithofacies holds significant importance in characterizing subsurface reservoirs. The lithology log, an essential segment of the master log, delineates the sequences encountered in subsurface drilling. This log offers a real-time depiction of the subsurface layers. Utilizing lithology logs proves valuable for correlating and comparing equivalent parts or subsections across various areas. Depending on the geologist's goals, these logs can differ in format and style. Their primary function is to display geological and lithological formations. A lithology log is a visual summary of underground sedimentary rock units. Summarising extensive data, identifying patterns, and recognizing changes in sedimentary facies due to creating an overview of the vertical sequence are some of the key benefits of such logs. Additionally, these logs are appropriate for verifying correlations across sections of the corresponding age in diverse regions, called well-to-well correlation^1^. In the geo-energy industry, accessing and analyzing lithology logs for reasons like the age of drilled wells and mud loss is challenging. In such cases, they are traditionally generated manually by visually correlating lithology logs from nearby wells. Subsurface geological heterogeneities exacerbate this technique's inaccuracy^2^. Due to its reliance on the interpreter's skills, the manual method has a relatively long processing time and has considerable generalization errors. Aside from that, even experienced interpreters find this method cumbersome and inefficient when dealing with the increasing volume of data.

Additionally, cross-plot characterization can categorize lithofacies from well logs. Typically, well logs are sampled continuously as part of underground exploration. Besides measuring the petrophysical characteristics of subsurface rocks, well logs facilitate understanding lithofacies by revealing lithology, texture, and structure changes. In light of the rising volume of data, cross-plot characterization also becomes time-consuming and challenging, even for skilled interpreters. Salinity, fluid content, diagenesis, fractures, and clay composition can exhibit parallel log reactions to lithology in standard well logs. Nevertheless, well-log patterns for distinct lithologies, notably their transition subtypes, can be identical. In cross plots, these cases can complicate and non-linearise the problem. The Exploration and Production industry has focused on machine learning (ML) techniques in light of their potential to handle non-linear issues, the massive volume of data, the need for skilled interpreters, and manual methods' generalization errors^3–10^. Developing an ML-based methodology to generate high-resolution lithology logs via conventional well logs and lithology logs from nearby wells may be crucial.

Over the past several decades, researchers have extensively investigated how ML techniques can identify lithofacies from well logs. Unsupervised learning techniques, e.g., expectation-maximization^11^, K-means clustering^12^, hierarchical clustering^13^, self-organizing map^14^, and deep autoencoder^15^, provide only an overall perspective by arranging the lithofacies based on their inherent characteristics. They are helpful in cases where the dataset is limited, i.e., no label is available. In contrast, semi-supervised learning techniques, e.g., positive and unlabeled ML^16^, active semi-supervised algorithms^17^, and laplacian support vector machine (SVM)^18^, are beneficial when a limited amount of labelled data is accessible. Conversely, the supervised learning technique is applicable when lithofacies are pre-defined in a well, and we need to determine which labels from the second well belong. Several well-known supervised shallow learning algorithms are traditionally employed for lithofacies classification based on well logs labelled by cores. This category encompasses backpropagation neural networks^19^, SVM^20^, bayesian networks^21^, K-nearest neighbor^22^, logistic regression (LR)^23^, decision tree (DT)^24^, kernel Fisher discriminant analysis^25^, quadratic discriminant analysis^26^, gaussian naive Bayes^27^, and bayesian-artificial neural network^28^. Moreover, homogeneous ensemble techniques, e.g., random forest (RF)^29^, adaptive boosting model^30^, extreme gradient boosting (XGBoost)^31^, gradient boost DT^32^, logistic boosting regression, and generalized boosting modeling^33^, also fall under the same category. Additionally, the integration of RF and XGBoost^34^, the combination of artificial neural networks and hidden Markov models^35^, and the stacked generalization of K-nearest neighbours, DT, RF, and XGBoost^22^ can be considered heterogeneous ensemble algorithms in the related domain. Such supervised algorithms use geological rules, making lithofacies estimation more trustworthy^3^. Moreover, researchers have employed several deep learning (DL) algorithms, e.g., convolutional neural networks (CNNs)^36^, hybrid CNN-long short-term memory networks^37^, and TabNet^38^, to classify lithofacies via core-labelled well logs. Nevertheless, many DL applications need to pay more attention to the significance of sample size, a critical factor for effective lithofacies modeling. Generally, a more complex problem demands more sophisticated and improved algorithms, which, in turn, request more training data. Collecting such a volume of data can take time and effort, making the process infeasible. To address the sample size dilemma in lithofacies classification tasks, transfer learning, which uses DL models trained on large amounts of data, has emerged as a solution^3^. Transfer learning, however, requires access to a large volume of data similar to or related to the upcoming problem dataset. It may be possible to locate such data sources occasionally, but this may only sometimes be true. Alternatively, ensemble learning involves combining several baseline models into a larger one with more robust performance than each model individually. Furthermore, combining diverse baseline models reduces overfitting risk in ensemble learning. Many fields and domains have benefited from ensemble learning, often outperforming single models^39,40^. The selection of baseline classifiers in ensemble techniques results in differences. Two methodologies, homogeneous and heterogeneous ensembles, generate multiple classifiers based on their structure. Homogeneous ensembles, e.g., RF and bagging^41^, comprise similar baseline classifiers that utilize different datasets. The major limitation of homogenous systems is generating diversity using a single algorithm. In contrast, the heterogeneous ensemble, e.g., voting^42^ and stacking^43^, consists of several baseline classifiers trained on a single dataset^44^. Research has proven that heterogeneity in base classifiers contributes to developing more accurate, robust, and scalable ensemble models^45^. Ensemble methods provide a means to handle non-linear, intricate, and multi-dimensional geoscience data^46,47^.

As aforementioned, to date, researchers have utilized several supervised shallow/deep algorithms to determine the correspondence among multiple varieties of well logs (as input) and lithofacies derived from core data or well logs (i.e., electrofacies) (as target) and then used the resultant correlation to locate lithofacies in uncorded intervals/wells. However, this research focuses on designing a robust and scalable heterogeneous ensemble-based workflow for lithofacies modelling using lithology logs as the target. Nevertheless, several significant drawbacks can be found in nearly all ML/ensemble-based paradigms for lithofacies classification, mainly (1) their scalability constraints and (2) their ignorance of multiclass imbalances in data. The investigation attempts to overcome the first drawback by utilizing the blind well dataset from an oilfield with bold geological heterogeneity. As the second drawback, subsurface geological heterogeneities place lithofacies modelling problems in the spotlight in various real-world scenarios with multiclass imbalanced data classification difficulties. Due to their focus on accuracy, traditional classifiers encounter challenges in performance when confronted with class imbalance, leading to neglect of the minority class or classes. Moreover, conventional ML algorithms such as SVM, primarily devised for binary classification tasks, often demand adjustments to attain optimal performance in multiclass scenarios^48^. Furthermore, most standard imbalanced data combat tactics, e.g., cost-sensitive learning (CSL)^49^, adaptive synthetic sampling (ADASYN), and modified synthetic minority oversampling technique (M-SMOTE) (as resampling techniques)^50^, are designed for binary issues and fail to adapt directly in situations with multiple classes. However, in some research, e.g., Liu and Liu^37^ and Zhou et al.^32^, imbalanced binary data combat tactics have been directly implemented for imbalanced multiclass lithofacies classification situations. We utilized decomposition techniques to extend imbalanced binary data combat tactics and binary-based ML algorithms (e.g., SVM) to multiclass environments. The original datasets are broken down into binary sets as part of these techniques by a divide-and-conquer procedure. Consequently, multiple classifiers are required, each responsible for a specific binary problem. Decomposition strategies are divided into two main categories, i.e., One-vs.-All (OVA) and One-vs.-One (OVO). When there are *k* classes in a problem, OVA compares each class with the others using $k$ binary classifiers. Alternatively, OVO uses $k(k-1)/2$ binary classifiers to differentiate between class pairs in $k$-class problems^3^. These binary classifier architectures can be significantly improved using error correcting output code (ECOC)^51^. Furthermore, by under-sampling the majority samples or over-sampling the minority observations, resampling techniques seek to balance data. Nevertheless, these methods will likely exclude some relevant information or even raise the processing rates of irrelevant samples. Under-sampling techniques (e.g., one-sided selection^52^) and over-sampling algorithms (e.g., borderline-synthetic minority oversampling^53^) alter class distribution. In return, CSL considers the costs of misclassifying samples^49^. Additionally, there are other options available in this situation besides class decomposition. This way, the research uses ad-hoc approaches designed to learn directly from dataset^54^.

In this study, we aim to develop a scalable ensemble-based workflow to generate high-resolution lithology logs reliably and automatically. We address two challenging topics: (1) the scalability of the designed workflow and (2) the analysis of the multiclass imbalanced dataset. The initial obstacle is overcome using a blind well dataset from an oilfield with complex heterogeneous conditions. Besides ad-hoc strategies, combining decomposition techniques with binary imbalance data combat tactics is crucial in addressing the second concern. In this investigation, a heterogeneous ensemble model is designed and compared with baseline classifiers as popular algorithms in lithofacies classification research.

## Methodology

### General workflow

**Figure 1:**
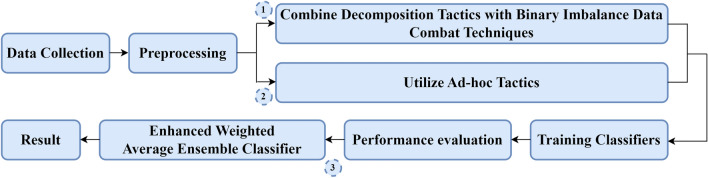
An overview of the proposed workflow.

Figure 1 demonstrates an overview of the proposed high-resolution lithology log generation workflow, consisting of three main subsections: Workflows 1, 2, and 3. Following data collection and preprocessing, it is partitioned into training, testing, and blind verification datasets. Workflow 1 evaluates the interaction of the baseline classifiers with the synergy of decomposition techniques and binary imbalanced data handling methods. Through Workflow 2, the baseline classifiers are coupled with ad-hoc approaches. Finally, after the training and evaluation all baseline classifiers, an enhanced weighted average ensemble of outstanding classifiers is integrated with superior synergies/ad-hoc tactics in Workflow 3.

### Multiclass imbalanced learning

Even though minority classes are rare, they frequently provide vital knowledge and crucial learning content. This section should address two main challenges: (1) the usability of standard ML algorithms and (2) the feasibility of conventional binary imbalance data combat tactics for solving multiclass imbalance issues. A widely accepted methodology to simultaneously address both obstacles involves dividing the multiple-class modelling issue into several binary subproblems through ECOC, OVA, and OVO as decomposition strategies. This investigation focuses on the ECOC encoding process due to its functionality (in contrast, OVO/OVA). Specifically, this is true regarding overlap due to the vicinity across classes' spectrum and influenced by their spatial positions. By exploiting ECOC, it is possible to use standard ML algorithms and strategies for combating binary imbalance data in the upcoming multiclass imbalance concern. However, several studies have concentrated on an overall framework that focuses on developing ad-hoc methods like Static-SMOTE^55^ instead of modifying conventional techniques for handling binary imbalance data in the multiclass context. Ad-hoc approaches are generally limited to several specific types of research and are not very general. Additionally, CSL can handle an imbalanced binary class^56,57^. CSL proves more effective than sampling techniques (e.g., M-SMOTE) for imbalanced varieties^58^. Unlike sampling methods, CSL maintains the original distribution of data^59^. As a result, due to CSL's capabilities, this paper focuses on its ability to address imbalanced data challenges. In the current research, through the ECOC technique, the existing imbalanced multiclass problem is decomposed into binary subsets. Then, strategies for dealing with imbalanced binary data are implemented to address it. Additionally, the study utilizes Static-SMOTE as an ad-hoc tactic to highlight the efficiency of the proposed technique.

#### Error correcting output code concept

Theoretically, encoding and decoding are the two phases involved in ECOC schemes. Encoding results in a confusion matrix, while decoding places every unidentified instance in the most similar class. An $N*m$ confusion matrix has a ${c}_{i,j}$ element in the *i*th row (${c}_{i}$) and *j*th column. The *i*th class and the *j*th column are respectively symbolized by ${cla}_{i}$ and ${col}_{j}$. The confusion matrix must meet five specifications simultaneously. Initially, every row ought to include either a ' + 1' or ' − 1':

1 $$\mathop \sum \limits_{j = 1}^{m} abs\left( {c_{i,j} } \right) \ne 0, \forall j \in \left[ {1, N} \right]$$

If not, the relevant class cannot be identified during training. Secondly, to provide training examples for each group, all columns must include a ' + 1' or ' − 1':

2 $$\mathop \sum \limits_{i = 1}^{N} abs\left( {c_{i,j} } \right) \ne abs\left( {\mathop \sum \limits_{i = 1}^{N} c_{i,j} } \right), \forall j \in \left[ {1, m} \right]$$

The third rule is to avoid having duplicate overlapping columns:

3 $$\mathop \sum \limits_{i = 1}^{N} abs\left( {c_{i,j} - c_{i,l} } \right) \ne 0, \forall j,l \in \left[ {1, m} \right], j \ne l$$

As a fourth rule, no two rows should be alike:

4 $$\mathop \sum \limits_{i = 1}^{m} abs\left( {c_{i,j} - c_{l,j} } \right) \ne 0, \forall i,l \in \left[ {1, N} \right], i \ne l$$

Lastly, no pair of columns should have a reverse correlation:

5 $$\mathop \sum \limits_{i = 1}^{N} abs\left( {c_{i,j} - c_{i,l} } \right) \ne 0, \forall j,l \in \left[ {1, m} \right], j \ne l$$

Every dichotomizer selects a random element $s0$ during the decoding process, which forms the decoded vector ${y}_{s0}$. Typically, hamming distance ($HD$) is applied to assess similarities among ${y}_{s0}$ with ${c}_{i}$, and $s0$ being allocated to the *cla*_*o*_ exhibiting the most similarities.

6 $$HD\left( {y_{s0} ,c_{i} } \right) = \mathop \sum \limits_{j = 1}^{m} \left( {1 - sign\left( {y_{s0,j} .c_{i,j} } \right)} \right)$$

7 $$o = argmin_{{i = \left\{ {1, \cdots ,N} \right\}}} HD\left( {y_{s0} ,c_{i} } \right).$$

In this case, ${y}_{s0,j}$ refers to the *j*th item in ${y}_{s0}$. In cases where soft outcomes are required, the euclidean distance ($ED$) is applied instead of $HD$, which is restricted to complex results (+ 1/ − 1):

8 $$ED\left( {y_{s0} ,c_{i} } \right) = \sqrt {\mathop \sum \limits_{j = 1}^{m} (y_{s0,j} - c_{i,j} )^{2} }$$

Data-independent and data-dependent strategies can be used to produce optimum confusion matrixes. The earlier method generates confusing matrixes without considering the samples' distribution. Subsets of this approach include OVA and OVO. Due to the predetermined nature of the confusion matrixes in this category, they cannot be used on a wide range of data sets with satisfactory results. In contrast, the latter method creates confusion matrixes considering the numerical distributions, of which Data-Driven ECOC is one of its categories. Due to the better fit of its confusion matrixes to sample distributions, it typically provides superior classification performance^60^.

#### Cost-sensitive learning method

In analyzing data, the CSL tactic refers to a learning approach considering misclassification costs. Total cost minimization is its objective. Under CSL procedures, such as the MetaCost approach, various classes pay varying costs to address class imbalance challenges. CSL can be used to handle the costs associated with unfair misclassifications and class imbalances. CSL consists of two distinct groups. Developing classifiers that are independently cost-sensitive constitutes the primary group. A "wrapper" is designed in the second group that converts current cost-insensitive classifiers to cost-sensitive ones^61^. Due to its ability to convert a wide range of cost-intensive classifiers to cost-sensitive ones, the present study applies an instance-based weighting scheme from the second group. Adjusting class weights is one of the most straightforward ways to increase the algorithm's sensitivity to minority class/classes (particularly in models that incorporate class weights). Logically, penalties for the misclassification of distinct categories correspond with class weights. A class with a higher weight will be subject to higher penalties for misclassification than classes with a lower weight. There are several options for setting the weight of classes. This investigation utilizes the following equation as a balanced heuristic for class weight determination:

9 $$w_{c} = \frac{N}{{\left( {k*\left| c \right|} \right)}}$$

where ${w}_{c}$ refers to the weight assigned to the class $c$, $N$ denotes the number of classes within the dataset, $k$ stands for the class count within the dataset, and $\left|c\right|$ represents the sample count for class *c*^62^.

*Baseline classifiers.* SVM, DT, RF, LR, and XGBoost are selected baseline classifiers. The selection of such algorithms was deliberate, aiming to leverage the diverse strengths of each model for addressing various aspects of the research problem. Indeed, a diverse array of baseline algorithms, including linear, non-linear, homogeneous ensemble, and tree-based methods, provides varied learning strategies for the available dataset. SVM handles complex boundaries well. It uses a hyperplane to divide n-dimensional attribute vectors into two classes. Kernel functions are utilized to train the SVM algorithm, facilitating the transformation of feature vectors into higher-dimensional domains. After that, the convex optimization approach is adopted to solve the ML task. According to the maximum marginal hyperplane, every incoming instance should fit logically into either of the categories. A support vector is a set of data points nearest the hyperplane, which divides the class^63^. Additionally, DT offers interpretability and enables analysts to create intelligent forecasting classifiers. A DT allows users to estimate an object's value based on gathered data. In light of a set of relevant decisions, DT illustrates potential scenarios. As a result of this approach, users can weigh various decision alternatives, the costs, the probability, and the importance of every option. This study implements a classification and regression tree training procedure. The procedure facilitates classification and regression tasks by utilizing discrete or contiguous parameters. Classification and regression trees have just a pair of leaves on each node^64^. The classification task could also be conducted using RF, which provides robustness through ensemble learning. The model generates multiple DTs (or a forest) for the training process. When performing classification tasks, the model returns the class that corresponds to the mode of classes. Moreover, this approach eliminates the risk of overfitting inherent in DTs^65^. LR is another ML algorithm primarily designed for predicting class membership, in which the objective is to estimate the probability of whether an instance falls into a particular class^66^. LR offers simplicity and is adequate for binary classification tasks. Moreover, XGBoost is a popular ML algorithm suitable for tabular data, ensuring high performance and scalability. With XGBoost, it is possible to detect complex numerical correlations between the measured parameters and the desired model. This method combines conventional regression and categorization trees alongside analytic boosting algorithms. XGBoost details are available at Raihan et al.^67^. Table 1 outlines the hyperparameters obtained through hyperparameter tuning for baseline classifiers. These specific parameters are carefully chosen following preliminary experiments and subsequent fine-tuning conducted through grid search and cross-validation. This iterative process aimed to attain optimal performance while mitigating the risk of overfitting.

**Table 1:** Hyperparameters of baseline classifiers.

Baseline classifier	Hyperparameters
SVM	Kernel: Radial Basis Function (RBF), C (Regularization Parameter): 8.0, Gamma: 0.001
DT	Criterion: Gini impurity, Max Depth: 5.0, Min Samples Split: 5.0
RF	Number of Estimators: 128.0, Max Depth: 8.0, Max Features: 'sqrt'
LR	Solver: 'liblinear', Regularization: L2, C (Regularization Parameter): 10.0
XGBoost	Number of Boosting Rounds: 100.0, Learning Rate: 0.1, Max Depth: 3.0, Objective Function: Binary logistic regression

#### Voting ensemble classifier

Voting ensembles combine estimates of several distinct classifiers. This technique improves the performance of individual classifiers in an ensemble, ideally outperforming any single algorithm. Pooling forecasts across different algorithms enables the creation of a voting ensemble applicable to regression and classification problems. During classification, estimates for each label are added together, and the majority vote label is determined. Suppose $\mathrm{N}$ classifiers are chosen and identified by ${\mathrm{S}}_{1},\dots , {\mathrm{S}}_{\mathrm{N}}$ and $\mathfrak{R}=\left\{{\mathrm{S}}_{\mathrm{i}}:\mathrm{i}=1, 2, 3, \dots \mathrm{N}\right\}$. In the case of $\mathrm{M}$ output classes, the ensemble voting algorithm determines how to combine the classifier ${\mathrm{S}}_{1}$ by voting $\mathrm{V}$ to optimize the $\mathrm{F}(\mathrm{V})$ function. An array with dimensions $\mathrm{N}\times \mathrm{M}$ represents $V$. An indication of the weight of *i*th classifier's vote for the *j*th class is provided by $\mathrm{V}\left(\mathrm{i},\mathrm{j}\right)$. As a general rule, the more confident a classifier is, the greater the weight allocated, while the more uncertain a classifier is, the lower the weight assigned. $\mathrm{V}\left(\mathrm{i},\mathrm{j}\right)\in \left[\mathrm{0,1}\right]$ represents the level of assurance the *i*th classifier has for the *j*th class. Combination rules use weights to combine the predicted outcomes of classifiers. There are two approaches to predicting the majority vote for classification: hard voting and soft voting. Hard voting involves calculating the total number of votes for each class label and predicting which has the most votes. Soft voting involves summing the probability estimates of each class label, and the predicted class label is the one with the highest probability. Voting ensembles are recommended when all models in an ensemble are predominantly in consensus or have similar exemplary performance. They are particularly beneficial whenever several fits of identical baseline classifiers are combined with various hyperparameters^68^. The voting ensemble is limited in considering all algorithms equally, i.e., each model contributes identically to forecasting. To address such issues, an extension of the voting ensemble involves applying weighted averaging or weighted voting of the collaborating algorithms.

#### Enhanced weighted average ensemble method

This paper applies the enhanced weighted average ensemble model^69^ to classify multiclass imbalanced data. These ensembles have shown their effectiveness, accuracy, reliability, and robustness in addressing complex pattern recognition challenges^70^. Baseline classifiers that are more skilled than others are given additional weight in this method. The algorithm modifies voting ensembles in which all models are deemed equally qualified and contribute identically to predictions. Each baseline classifier is assigned a weight to determine its contribution amount. Finding appropriate weights is a challenge for such algorithms. Optimum weights result in superior efficiency to ensembles based on similar weights and individual baseline classifiers. The present study utilizes the Grid Search strategy, assigning weights from a range of [0.0, 0.1, 0.2, 0.3, 0.4, 0.5, 0.6, 0.7, 0.8, 0.9, 1.0] to each baseline classifier. This approach aims to optimize the assigned weights effectively, addressing the challenge. Additionally, the research utilizes soft and hard estimators for voting.

### Case study

One of the Middle East oil fields is selected as a case study. Geologically, the field lies in the transition zone between the highly folded Zagros region and the stable Arabian platform. The underground formations explored are Gurpi, Ilam, Laffan, Sarvak, and Kazhdumi, whose predicted strata are as follows:

1. The Gurpi Formation comprises a sequence of Shale (Sh), Limestone (Ls), and Argillaceous Limestone (argiLs) stratigraphically associated with the Ilam Formation (at the top section).
2. The Ilam Formation is composed mainly of yellow to grey-brown Ls containing glauconite alongside trace quantities of hydrocarbons. Oolitic Ls appear frequently intermingled with Ls. There are traces of Sh segments in its lower part and evidence of hydrocarbons. Sh sequences, secondary Ls, and hydrocarbon remains are in the top position.
3. There are greyish to emerald ash Sh layers with fine inclusions of white Ls in the Laffan Formation (roughly 10 m thick).
4. The Sarvak Formation's lower lithotype contains numerous Sh layers and hydrocarbon residues. In the remainder, there are predominantly grey Chalky Limestones (chkLs), light grey to white chkLs, and dark brown to pale brown Cherty Ls. Regional Sh accompanies these Lss.
5. Kazhdumi Formation generally consists of dark black and dark brown Sh and pyritic Ls, rich in dark grey to pale ash and dark brown Sh-Ls.

### Dataset

**Figure 2:**
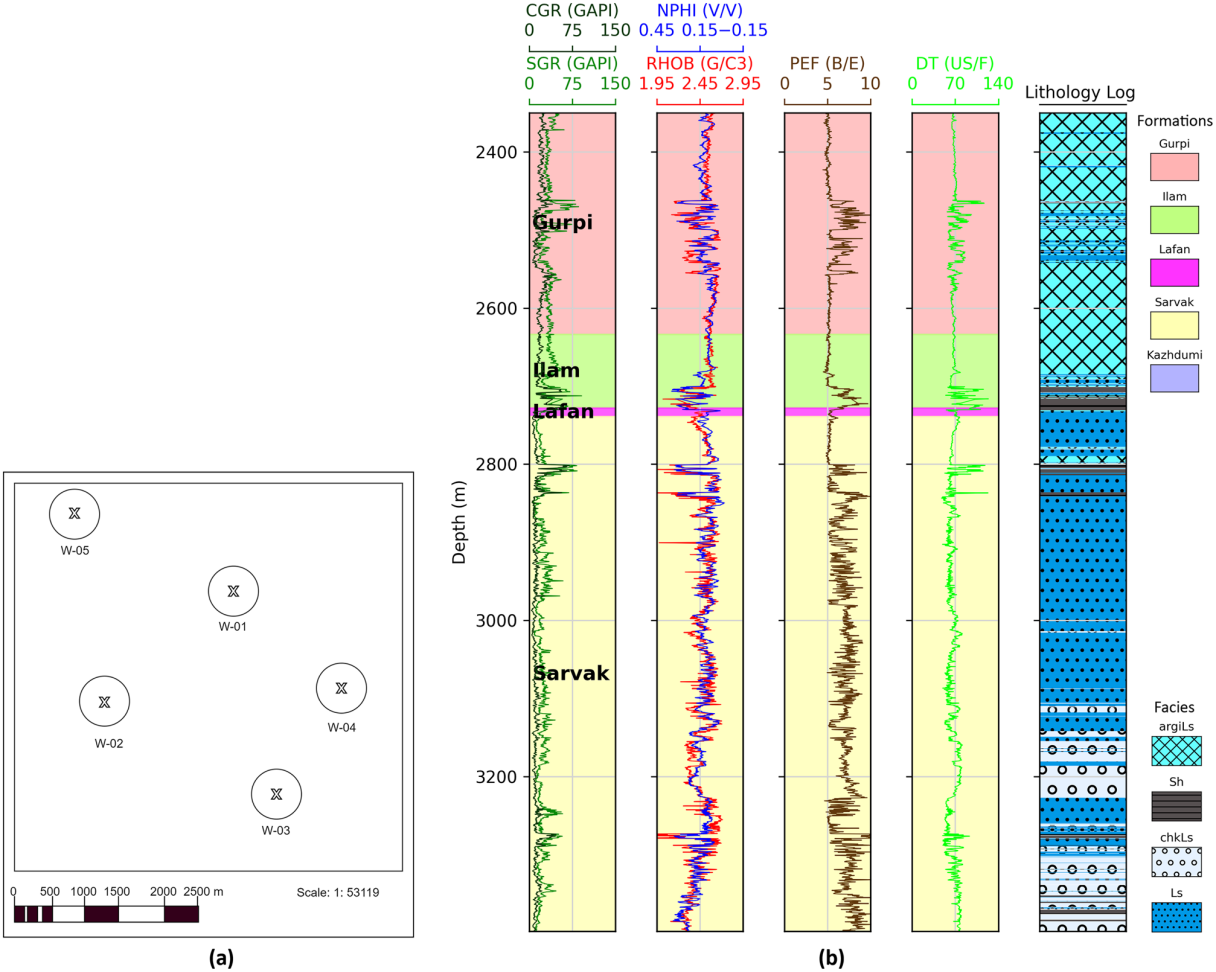
(**a**) The geographic positions of the wells in the area under investigation, and (**b**) Conventional well logs, lithology log, and a legend map for W-02 as an illustrative example.

**Figure 3:**
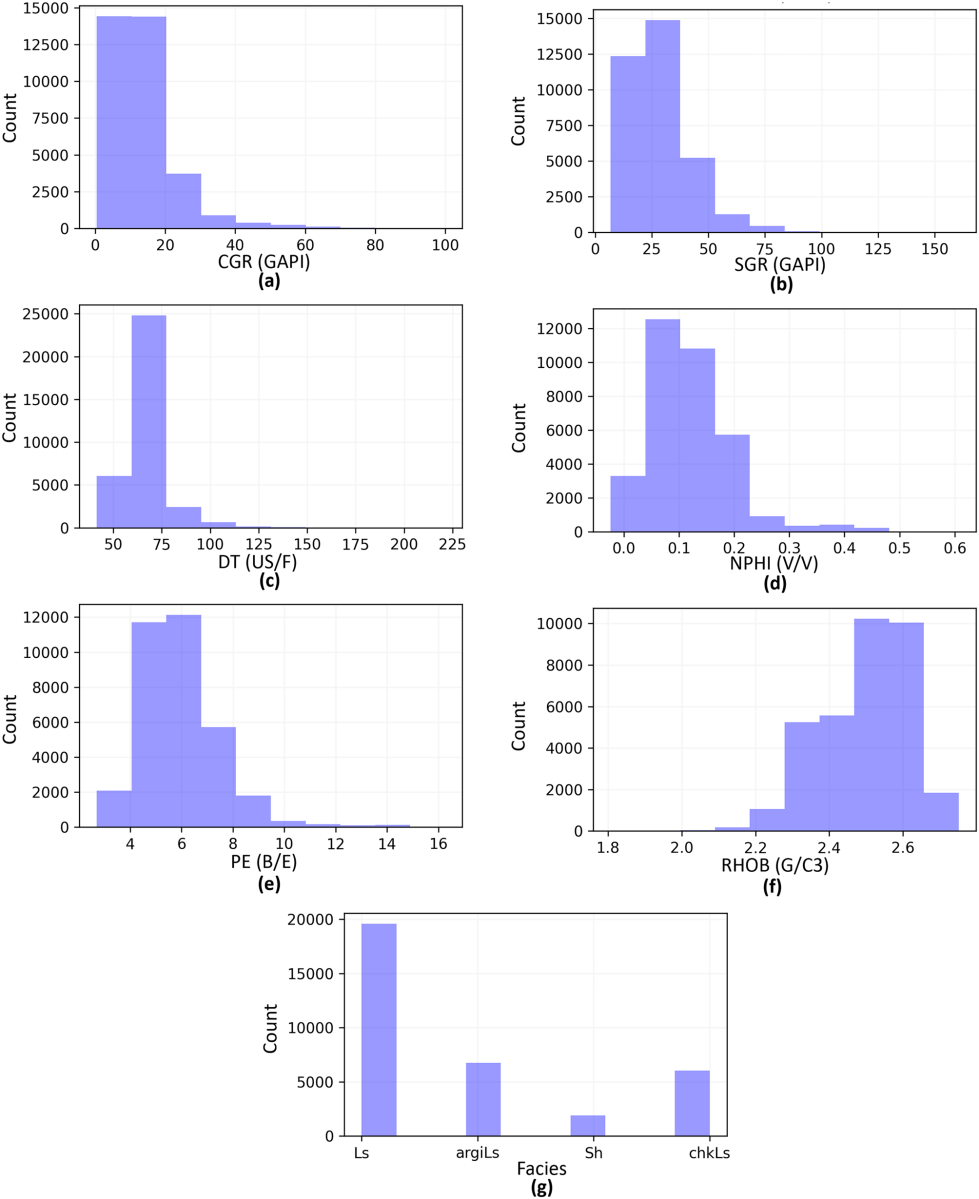
Distribution of input features including (**a**) CGR, (**b**) SGR, (**c**) DT, (**d**) NPHI, (**e**) PE, and (**f**) RHOB, alongside (**g**) Facies as the target feature.

The dataset consists of computed gamma ray [CGR (GAPI)], spectral gamma ray [SGR (GAPI)], neutron porosity [NPHI (V/V)], photoelectric factor [PE (B/E)], density [RHOB (G/C3)], Sonic [DT (US/F)], and lithology logs. Data from five wells identified as W-01 to W-05 exist within the study area. Figure 2a demonstrates the geographical positions of the wells in the area under investigation. W-03 is selected as a blind well based on its geographical location and data range coverage. The ML algorithms are trained using data from the other four wells. For instance, Fig. 2b illustrates the conventional well logs and lithology logs for W-02. Figure 3a–g display the distribution of input features (CGR, SGR, DT, NPHI, PE, RHOB) and target features (Facies), respectively. Figure 3g illustrates a substantial imbalance within the input data.

### Data preparation and class differentiation

As a part of this subsection, the data undergo a check for missing values and outliers after encoding categorical features (such as facies names, well identifiers, and formations) into dummy variables. An error in a dataset can take many forms, for example, duplicate rows or weak columns. While refining the available data, columns with only a single value, low variance, and rows containing repeated observations are identified and eliminated. Additionally, unnecessary columns are eliminated based on the correlation between different features. Furthermore, the distribution quantity of available datasets necessitated the application of standardization. Before presentation as input to the ML algorithms, the data undergo standardization to achieve a zero mean and unit variance^71^. However, complications like drilling fluid disturbance or drill bit balling up during lithology log recording can occur. Therefore, it could be challenging to separate different facies because of these bugs. Before training the classifier, the preprocessing stage aims to achieve a high level of separation between other classes. This goal is performed using linear discriminant analysis as a noise reduction technique^72^ with 97% accuracy. By stratifying sampling^73^, the input data are divided between training (75%) and testing (25%) to account for the problem of data imbalance. Thus, both sets have a proportional representation of class.

## Results and discussion

The study initiates with Workflow 1 (see Fig. 2), aimed at assessing the baseline classifiers while exploring synergies between the decomposition strategy and various tactics tailored for handling imbalanced binary data. This phase is crucial for pinpointing noteworthy interactions. Furthermore, Workflow 2 amalgamates optimal baseline classifiers with customized ad-hoc methods. Subsequently, Workflow 3 introduces an enhanced weighted average ensemble that merges the most effective baseline classifiers. This ensemble is then integrated with superior synergies or ad-hoc techniques for an improved performance assessment. The assessment of imbalanced multiclass classification presents a challenge because widely used measures for evaluating classifiers' outputs, such as accuracy, are built upon assumptions of balanced distributed data. Previous studies have proposed Mean Kappa statistics (*Mean. K*) and Mean F-measures (*Mean. F*) to assess imbalanced situations^74–76^. The Landis and Koch grouping is commonly utilized for interpreting Kappa statistics values, where the ranges correspond to different levels of agreement: 0% (poor); 0–20% (slight); 21–40% (fair); 41–60% (moderate); 61–80% (substantial); and 81–100% (almost-perfect)^77^. For a detailed explanation of the Kappa statistic and F-measure for imbalance multiclass classification, refer to Jamshidi Gohari et al.^3^. Developing lithology log generation within the Google Collaboratory platform involves various libraries. These libraries include Pytorch, Pandas, Numpy, Matplotlib, Mpl toolkits, and Sklearn in Python 3.11.5. Additionally, we ran on an Intel Core i7-11370H with 16 GB of RAM.

### Synergy between ECOC and binary imbalanced data combat tactics

This subsection through Workflow 1 describes how ECOC and binary imbalanced data combat tactics interact with baseline classifiers. As part of Workflow 2, Static-SMOTE highlights the results. Table 2 illustrates average outcomes and rankings based on the average of 20 runs. The *t*-index represents test marks, whereas the *b*-index indicates blind evaluation scores. One section covers the ad-hoc approach, and the other presents the ECOC scheme. Each technique is ranked separately for a given unit in the "*Rank*" column. The highest marks are indicated in bold font. Furthermore, the basic version of the algorithms (i.e., Base and Std) is implemented to verify the results. Table 2 supports the following findings. When combined with ECOC and CSL as a corporator of Workflow 1, SVM produced the most accurate results (*Rank*_*b*_ = 1). The effectiveness of this procedure manifested itself in a *Mean. F*_*b*_ of 86.87% and a *Mean. K*_*b*_ of 78.04% for blind well datasets. ECOC-CSL is numerically better than ECOC-M-SMOTE or Static-SMOTE. In addition, coupling RF with the synergy of ECOC and CSL yielded a *Mean. F*_*b*_ of 86.28% and a *Mean. K*_*b*_ of 77.29% as a co-factor of Workflow 1 (*Rank*_*b*_ = 2). In this particular combination, when paired with RF, ECOC-CSL demonstrates superior numerical performance compared to other methods, thereby affirming its overall functionality. When examining ECOC-CSL-SVM (*Rank*_*b*_ = 1) and ECOC-CSL-RF (*Rank*_*b*_ = 2) outputs, it becomes apparent that the former exhibits a higher level of proficiency. However, both perform satisfactorily on blind well data evaluation. Therefore, improving performance by developing an enhanced weighted average ensemble that combines these two synergies from Workflow 1 may result in superior performance.

**Table 2:** Mean classifier test and blind well assessment outcomes (using a 20-run average) for baseline classifiers based on *Mean. F* and *Mean. K* (Percentage-wise).

Method	Baseline classifier	Adaptation	${\mathrm{Mean}.\mathrm{ F}}_{\mathrm{t}}$	${\mathrm{Mean}.\mathrm{ F}}_{\mathrm{b}}$	${\mathrm{Rank}}_{\mathrm{b}}$	${\mathrm{Mean}.\mathrm{K}}_{\mathrm{t}}$	${\mathrm{Mean}.\mathrm{K}}_{\mathrm{b}}$	${\mathrm{Rank}}_{\mathrm{b}}$
Ad-hoc	SVM	Base	93.26	82.46	–	88.15	70.61	–
	RF		92.72	81.88	–	87.49	69.96	–
	XGBoost		90.62	78.74	–	84.97	67.54	–
	DT		88.54	76.65	–	82.65	65.89	–
	LR		84.38	71.84	–	77.86	60.85	–
	SVM	Static-SMOTE	93.33	83.58	5	89.24	72.55	5
	RF		92.58	82.75	6	88.43	71.69	6
	XGBoost		89.98	81.42	8	85.68	69.14	8
	DT		88.99	80.68	10	83.45	67.82	10
	LR		85.04	76.11	13	78.24	62.74	13
ECOC	SVM	Std	93.87	85.30	–	90.03	75.03	–
	RF		92.84	84.29	–	89.12	74.08	–
	XGBoost		89.76	83.02	–	87.45	72.88	–
	DT		87.65	81.45	–	85.94	70.86	–
	LR		82.98	77.07	–	80.85	65.87	–
	SVM	M-SMOTE	89.92	81.38	9	83.56	68.82	9
	RF		88.97	80.24	11	81.75	67.03	11
	XGBoost		86.43	77.54	12	78.54	64.72	12
	DT		83.95	72.97	14	77.14	62.68	14
	LR		80.87	71.95	15	72.56	57.21	15
	SVM	CSL	**94.71**	**86.87**	**1**	**91.37**	**78.04**	**1**
	RF		94.09	86.28	2	90.55	77.29	2
	XGBoost		93.87	84.08	3	89.62	75.42	3
	DT		93.74	83.67	4	89.48	74.14	4
	LR		90.32	81.54	7	85.98	70.52	7

The t-index signifies test grades, while the b-index denotes ratings from blind evaluations.

### SVM-RF enhanced weighted average ensemble development

**Figure 4:**
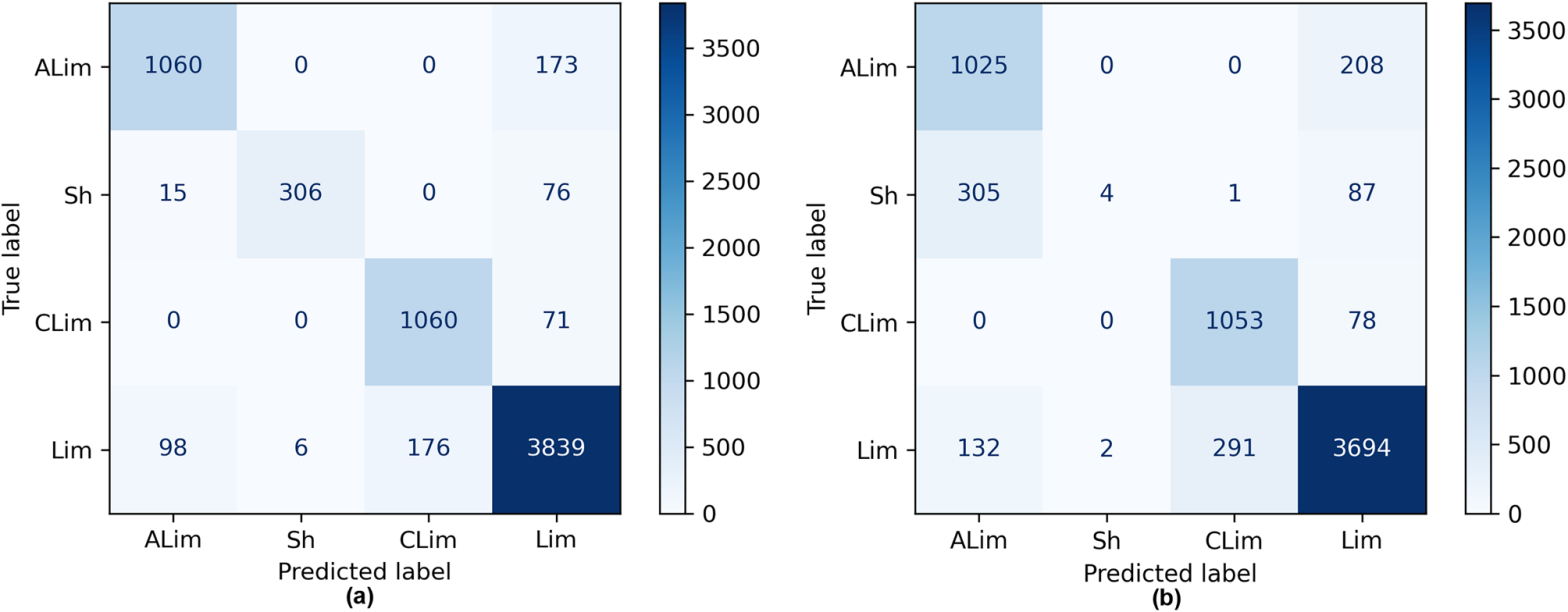
(**a**) Confusion matrix of the optimal workflow for blind well data evaluation, and (**b**) confusion matrix of an unoptimized workflow for blind well data assessment.

In this subsection, the development of an enhanced weighted average ensemble based on two superior combinations of Workflow 1, i.e., ECOC-CSL-SVM and ECOC-CSL-RF, is reported. The voting scheme consists of two types: soft voting and hard voting. Table 3 presents the average results and rankings across 20 runs. As reported, Workflow 3 provides the best performance, in which the enhanced weighted average ensemble of SVM and RF in soft voting mode is coupled with ECOC-CSL—a *Mean. F*_*b*_ of 91.04% and a *Mean. K*_*b*_ of 84.50%, which indicates almost perfect agreement, is proof of this superiority (*Rank*_*b*_ = 1). Tables 2 and 3 illustrate that the enhanced weighted average ensemble of SVM and RF in soft voting mode coupled with ECOC-CSL performs the most efficient workflow, henceforth called optimal workflow. Additionally, by comparing the confusing matrixes of the various workflows (i.e., Workflows 1, 2, and 3), the optimal workflow provided the superior prediction for argiLs, chkLs, Ls, and Sh. Figure 4a,b present the confusing matrixes comparing the optimized workflow against an unoptimized approach for evaluating blind well data. It's apparent that the unoptimized workflow exhibits bias towards the majority classes and performs suboptimally in recognizing the minority class, specifically Sh.

**Table 3:** Mean classifier test and blind well results (using a 20-run average) for designed ensemble based on *Mean. F* and *Mean. K* (Percentage-wise).

Method	ensenble type	Adaptation	${\mathrm{Mean}.\mathrm{ F}}_{\mathrm{t}}$	${\mathrm{Mean}.\mathrm{ F}}_{\mathrm{b}}$	${\mathrm{Rank}}_{\mathrm{b}}$	${\mathrm{Mean}.\mathrm{K}}_{\mathrm{t}}$	${\mathrm{Mean}.\mathrm{ K}}_{\mathrm{b}}$	${\mathrm{Rank}}_{\mathrm{b}}$
ECOC	Enhanced weighted average ensemble of SVM and RF in soft voting mode	CSL	94.92	91.04	1	91.70	84.50	1
	Enhanced weighted average ensemble of SVM and RF in hard voting mode		94.07	90.33	2	90.44	83.62	2

The t-index signifies test grades, while the b-index denotes ratings from blind evaluations.

### Graphical comparative assessment

**Figure 5:**
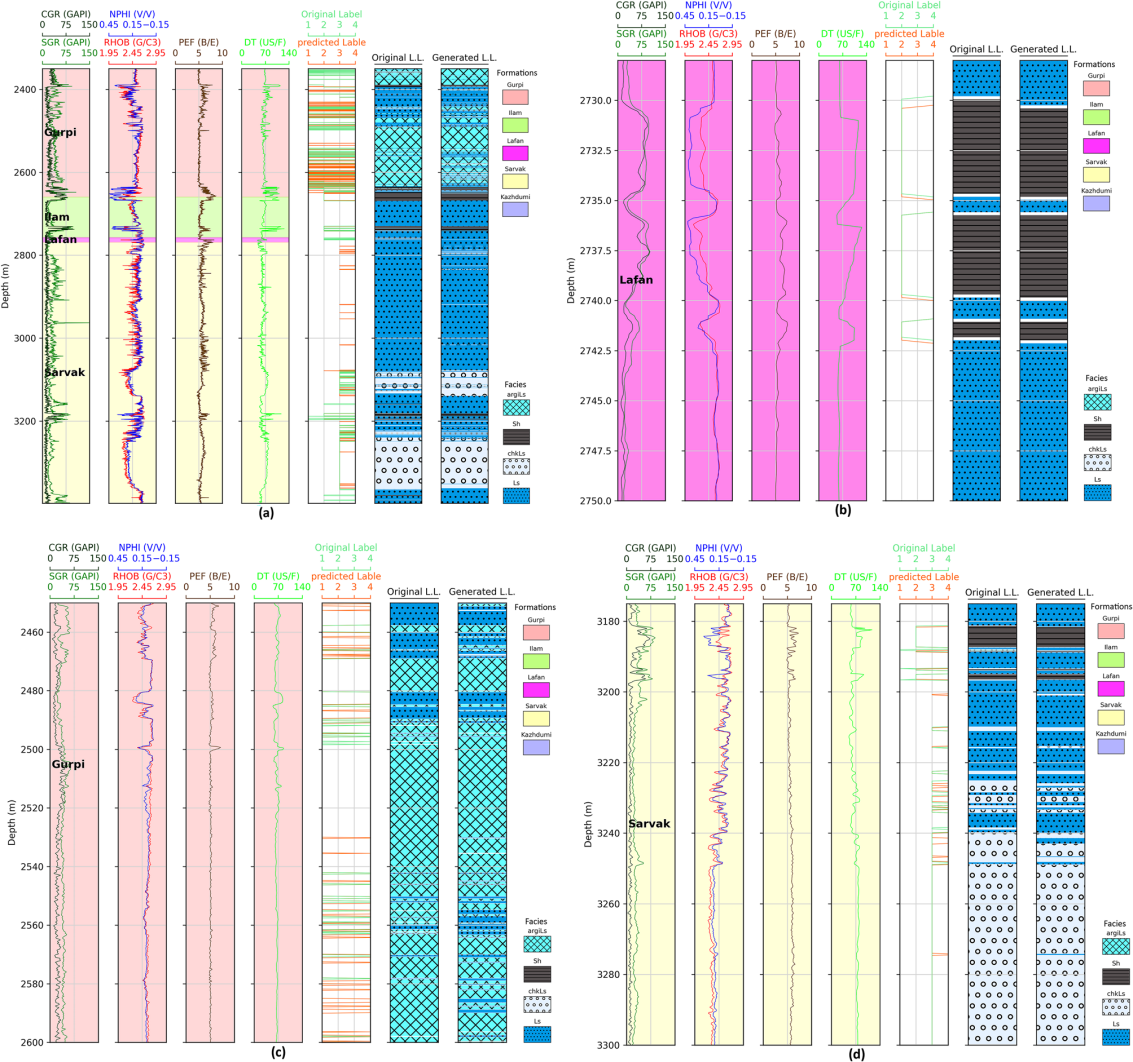
Lithology log (LL) generated using the optimal workflow for blind well data, illustrating depth intervals: (**a**) 2351–3399 m, (**b**) 2728–2750 m, (**c**) 2450–2600 m, and (**d**) 3175–3300 m.

Figure 5a–d, depict the generated lithology log (i.e., Generated LL) for different depth intervals through the optimal workflow from the blind well dataset. The optimal workflow could separate Sh as one of the critical minority classes from argiLs, chkLs, and Ls according to the peak values in the conventional well logs, especially CGR and SGR. The generated lithology log displays a reasonable similarity to the original one (i.e., Original L.L. in Fig. 5a–d) in pinpointing the regions where argiLs, chkLs, Ls, and Sh occur. Figure 5b displays the concentrating depth interval (2728–2750 m) for the minority Sh class in the blind well. It shows an excellent correlation among the peak positions of the blind well logs, the Sh positions in the original lithology log, and the generated one. A similar agreement holds to argiLs, chkLs, and Ls facies, which share overlapping characteristics. Figure 5c highlights the blind well interval of 2450–2600 m, covering the argiLs, and Ls facies. Additionally, Fig. 5d shows the depth interval of the blind well for chkLs, Ls, and Sh facies from 3175 to 3300 m. In these figures, the positions of argiLs, chkLs, Ls, and Sh in the generated lithology log reasonably match those in the original one.

Unlike the OVA and OVO approaches, which partition a multiclass modelling problem into a finite number of binary classification tasks, the ECOC algorithm allows any given class to be encoded as an infinite number of binary classification tasks. Excessive representation enables the additional models to function as "error-correction" forecasts, enhancing prediction ability. Furthermore, a significant factor that leads to superior CSL performance is assigning additional weight to misclassifications of minorities and imposing a penalty for inaccurate classifications. Thus, these classes are given more attention by the model. This approach compels the model to learn instances from minority classes, making it a potent tool for forecasting occurrences from these classes. CSL, on the other hand, maintains the original distribution of data, unlike resampling approaches. Moreover, the SVM classification effectiveness can be attributed to the fact that it transforms the initial data into a multi-dimensional space. This ability will separate the classes better while maintaining the exact computational cost as the initial problem. This feature is referred to as a kernel trick.

Furthermore, RF can minimize the impact of an imbalanced sample distribution during classification. This characteristic can enhance minority samples' identification efficiency. On the other hand, when the ratio of imbalanced observations rises, the classification performance of RF is markedly impaired. Due to this issue, it's not possible to train a complete classification algorithm. The current study addressed this drawback by coupling the RF with the ECOC-CSL. SVM behaved more skillfully than RF under similar conditions (i.e. when combined with the synergy of ECOC-CSL); however, both performed satisfactorily on blind well data evaluation. Designing an enhanced weighted average ensemble aims to maximize efficiency by combining these two models, each with unique advantages. As a result of its reduced rate of error and lower variance, the ensemble has an improved predictive performance over the individual models (i.e., baseline classifiers). However, to obtain optimum estimates, a unique classifier can only represent some of the fundamental characteristics of the data. Consequently, combining several primary learners can capture further insight into the data's internal layout and dramatically boost estimation precision.

In addition, the study seeks to offer a scalable workflow to generate lithology logs or, more broadly, to model lithofacies, not only restricted regions under investigation. Accordingly, the experiment sought to remedy conventional procedures' deficiencies and considered multiple factors. Hence, the research site with considerable geological heterogeneity was chosen, highlighting the imbalanced multiclass data issue. The optimal workflow performed superior results in the blind well evaluation. Therefore, it is confirmed through blind well analysis, another indicator of its scalability. Furthermore, given that geological evidence is based on lithology log data, it is crucial to consider its uncertainty sources. Wellbore instabilities (e.g., breakouts and washouts), balling up, and rheology disturbances can lead to inaccurate data sources. Incorporating LDA as a denoising tool to mitigate these concerns is advisable.

Additionally, the developed strategies for dealing with the multiclass imbalance dilemma manifest uniform performance irrespective of the classifier type. Consequently, the outcomes are comparable throughout, supporting validity. Finally, the DL algorithm is more stable than the shallow ML technique, particularly when analyzing noisy and uncertain geoscience datasets. As a result, it is recommended that the geoscience and geo-energy communities collect a global data bank similar to that developed in image processing to facilitate transfer learning. Moreover, this investigation primarily focused on several standard imbalanced data combat tactics and ad-hoc techniques. However, considering further alternatives, such as employing tailored loss functions like balanced cross-entropy and focal loss^78^ for imbalanced lithofacies modelling, is suggested as a reasonable avenue for future research directions. Last but not least, this study provides a basis for future work in geosciences and engineering that deals with multiclass data with imbalances.

## Conclusion

The current investigation focused on statistically and graphically analyzing high-resolution lithology log generation. A primary emphasis was placed on addressing two significant challenges: multiclass imbalance data classification and scalability. Three distinct workflows were scrutinized to tackle the former, employing baseline classifiers, a custom ensemble algorithm, and methods tailored for handling multiclass imbalance data. Addressing the latter challenge involved evaluating these workflows using blind well data from an oilfield characterized by substantial geological variations. The optimal workflow emerged as an enhanced weighted average ensemble of SVM and RF alongside ECOC and CSL. This amalgamation showcased notable strength and reliability, evidenced by a mean Kappa statistic of 84.50%, signifying almost-perfect agreement, and mean F-measures of 91.04%. These results underscore the optimal workflow's robustness and efficacy in evaluating blind well data. Moreover, the devised ensemble showcased superior performance to commonly employed baseline classifiers in lithofacies classification endeavours. This constructed workflow adeptly handles multiclass imbalanced data with efficiency and logical coherence. Evaluation based on statistical and graphical analyses of the blind well dataset indicated a satisfactory correlation between the generated lithology log and the original one. Additionally, a notable advantage of the proposed workflow lies in its ability to retain the initial data distribution. In summary, the developed workflow presents a versatile solution capable of addressing multiclass imbalance issues within the geo-energy sector, extending beyond lithofacies classification tasks.

## Data Availability

The corresponding author will make all the data available upon a reasonable request.

## Abbreviations

ML: Machine learning
CSL: Cost-sensitive learning
ADASYN: Adaptive synthetic sampling
ECOC: Error correcting output code
SVM: Support vector machine
RF: Random forest
DT: Decision tree
LR: Logistic regression
XGBoost: Extreme gradient boosting
CNN: Convolutional neural networks
DL: Deep learning
M-SMOTE: Modified synthetic minority oversampling technique
OVA: One-vs.-All
OVO: One-vs.-One
Sh: Shale
Ls: Limestone
argiLs: Argillaceous limestone
chkLs: Chalky limestones
CGR: Computed gamma ray log
SGR: Spectral gamma ray log
NPHI: Neutron porosity log
RHOB: Density log
PE: Photoelectric log
DT: Sonic log
$$HD$$: Hamming distance
$Mean. K$: Mean kappa statistics
$$Mean. F$$: Mean F-measures
L.L.: Lithology log
$$\mathrm{ED}$$: Euclidean distance
$$\mathrm{F}(\mathrm{V})$$: Voting function

## Subscript and superscript

*C*_*i,j*_: Number of confusion matrix elements
$$s0$$: Random element
$${y}_{s0}$$: Decoded vector
${w}_{c}$: The weight assigned to class $c$
